# Histological prevalence of *Helicobacter pylori* in patients undergoing upper gastrointestinal endoscopy at Cayenne Hospital and follow-up outcomes

**DOI:** 10.3389/fmed.2026.1785533

**Published:** 2026-04-28

**Authors:** Amandine Allard, Marthe Alogo A Nwatsok, Paul Ngock Dime, Dominique Louvel, Inès Fouoking Zomene, Corine Fozeu, Hedia Mahjoub, Florence Atodjinou, Amira Zerouali, Mathieu Nacher, Kinan Drak Alsibai, Alolia Aboikoni

**Affiliations:** 1 Service d’Hépato-Gastroentérologie, CHU de Guyane, Cayenne, French Guiana; 2 Centre d'Investigation Clinique (Inserm 1424), Institut Santé des Populations en Amazonie (ISPA), CHU de Guyane, Cayenne, French Guiana; 3 Université de Guyane, Cayenne, French Guiana; 4 Service d’Anatomie et Cytologie Pathologique, CHU de Guyane, Cayenne, French Guiana; 5 Registre des Cancers de Guyane (RCan Guyane), Institut Santé des Populations en Amazonie (ISPA), CHU de Guyane, Cayenne, French Guiana

**Keywords:** endoscopy, follow-up, French Guiana, *Helicobacter pylori*, histology

## Abstract

**Introduction:**

*Helicobacter pylori* (*H. pylori*) infects approximately half of the world’s population and is more prevalent in developing countries. It is a major risk factor for gastric cancer, which ranks fifth worldwide in both incidence and mortality. French Guiana is a French overseas territory located in the Amazon region and presents several characteristics that may favor *H. pylori* transmission compared with mainland France, including immigration from neighboring low-income countries, widespread socioeconomic vulnerability, a limited number of specialized healthcare professionals. In addition, the incidence of gastric cancer is high in this territory. However, the prevalence of *H. pylori* infection in this territory remains unknown. The aim of this study was to determine the histological prevalence of *H. pylori* infection in gastric biopsies, describe the clinical and histological characteristics of infected patients, and assess follow-up outcomes after diagnosis.

**Patients and methods:**

A retrospective study was conducted at Cayenne Hospital from January to December 2023 among patients with histologically confirmed *H. pylori* infection.

**Results:**

*Helicobacter pylori* was detected in 570 of 1,664 patients (34.3%). Among these, 545 infected patients were included in the analysis, of whom 57% were women. The mean age was 49.5 years. Two-thirds of the patients were foreign-born. The main indications for upper gastrointestinal endoscopy were epigastric pain (53%), gastroesophageal reflux disease (20%), and abdominal pain (19%). Histological examination revealed gastric adenocarcinoma in 1.65% of cases and gastric precancerous lesions in 28.4%, including 6.6% severe lesions requiring endoscopic surveillance. Regarding follow-up, only 52% (95% CI 47.8–56.3) of patients were aware of their diagnosis and 48% (95% CI 44.7–53.2) received eradication therapy, while 19% (95% CI 15.8–22.6) were lost to follow-up.

**Conclusion:**

The histological prevalence of *H. pylori* infection was 34.3%. Despite the presence of gastric precancerous lesions in nearly one-third of patients, post-diagnosis follow-up remained inadequate.

## 1 Introduction

*Helicobacter pylori* (*H. pylori*) infection is one of the most common chronic bacterial infections worldwide. It has well-established oncogenic potential and is a major risk factor for gastric cancer, which ranks fifth worldwide in both incidence and mortality (1–7). *H. pylori* infection is strongly associated with socioeconomic vulnerability and poor living conditions, as reflected by its high prevalence in developing countries (2, 8).

French Guiana is a French overseas territory located in the Amazon region of South America and presents several characteristics that may favor *H. pylori* transmission compared with mainland France. Immigration from neighboring low-income countries is substantial, and socioeconomic vulnerability is widespread (9, 10). These conditions are likely to promote the transmission of *H. pylori* and may contribute to a high prevalence of infection in this territory. Consistent with this hypothesis, the incidence of gastric cancer is relatively high in French Guiana (11, 12). In addition, access to healthcare and long-term patient follow-up can be challenging due to geographic constraints and a limited density of specialized healthcare professionals (13).

Despite these factors suggesting a potentially high prevalence of *H. pylori* infection, no studies have yet been published on *H. pylori* infection in French Guiana.

The aim of this study was therefore to estimate, for the first time, the prevalence of *H. pylori* infection based on histological examination of gastric biopsies obtained during upper gastrointestinal endoscopy (UGE), to describe the clinical and histological characteristics of infected patients, and to assess follow-up outcomes after diagnosis.

## 2 Patients and methods

A single-center retrospective study was conducted at Cayenne Hospital from January to December 2023. The study analyzed the histological findings of gastric biopsies obtained during upper gastrointestinal endoscopy (UGE). We estimated the proportion of *Helicobacter pylori* infections detected in these biopsies. The number of patients with *H. pylori*–positive gastric biopsies and the total number of patients who underwent gastric biopsy for histological examination were extracted from the pathology laboratory database to calculate the histological prevalence.

For descriptive analyses and follow-up assessment, patients with positive biopsies were included. Patients younger than 18 years, deceased patients, and those with incomplete medical records were excluded.

### 2.1 Histological methods

During upper gastrointestinal endoscopy, gastric biopsies were routinely obtained, including two biopsies from the antrum and one from the angulus, as well as at least two biopsies from the fundus and corpus. All specimens were sent to the pathology laboratory for histological analysis.

Formalin-fixed, paraffin-embedded gastric biopsy specimens were sectioned at a thickness of 3–4 μm using an HM 355S microtome (Microm Microtech, Brignais, France). Serial sections were mounted on glass slides, deparaffinized, and routinely stained with hematoxylin–eosin–safran (HES) for histopathological evaluation. For the detection of *Helicobacter pylori*, immunohistochemistry (IHC) was systematically performed on all biopsy specimens using a monoclonal anti–*Helicobacter pylori* antibody (NCL-L-Hpylori, Novocastra, Leica Biosystems, Newcastle, UK; dilution 1:100). Immunostaining was performed using an automated Bond-Max immunostainer (Leica Biosystems) according to the manufacturer’s protocol, including antigen retrieval and a polymer-based detection system. The immunostained slides were examined under light microscopy by an experienced pathologist. The presence of *H. pylori* was determined by identifying curved or spiral bacilli adherent to the gastric epithelium or located within the mucus layer.

Histological findings were interpreted according to the recommendations of the revised Sydney classification.

### 2.2 Histological findings

We evaluated histological features to identify precancerous gastric lesions, namely atrophy, intestinal metaplasia, and dysplasia. Atrophy was classified as mild, moderate, or severe. Intestinal metaplasia was classified as focal or extensive, with the latter including moderate and severe metaplasia. Dysplasia was classified as low-grade or high-grade.

The OLGA (Operative Link for Gastritis Assessment) and OLGIM (Operative Link on Gastric Intestinal Metaplasia Assessment) staging systems are based on the severity and topography of chronic atrophic gastritis and intestinal metaplasia, respectively. These staging systems are used to stratify the risk of gastric cancer and to guide the frequency of endoscopic surveillance. Patients classified as OLGA or OLGIM stage 3–4 have a higher risk of gastric cancer than those classified as stages 1–2 (14).

The OLGA and OLGIM stages were not routinely reported in the histopathological reports but were retrospectively estimated for all patients using the available histological data.

Among the precancerous lesions—including atrophy, intestinal metaplasia, and dysplasia—we defined a subgroup of “severe precancerous lesions,” corresponding to lesions associated with the highest risk of gastric cancer and requiring endoscopic surveillance (dysplasia and OLGA or OLGIM stages 3–4).

### 2.3 Clinical characteristics

For upper gastrointestinal endoscopy indications, the reasons documented in the consultation report were collected. We distinguished between abdominal pain and epigastric pain: when the location of the pain was specified as epigastric, the indication was recorded as “epigastric pain,” whereas when the location was not specified, it was recorded as “abdominal pain.”

For follow-up assessment, medical records were reviewed. When follow-up information was not available in the medical records, patients were contacted by phone or email according to the usual procedures of the Hepato-Gastroenterology Department. Physicians in the department routinely attempt to contact patients diagnosed with *H. pylori* infection by phone or email; however, this procedure is not exhaustive.

Patients were considered to be aware of their diagnosis if this information was documented in the medical records or reported during the phone call. Patients were considered to have been prescribed eradication therapy if a prescription was recorded in their medical records or if they reported receiving a prescription from a physician outside the hospital during the phone call. The same approach was used to determine whether a urea breath test had been prescribed.

Patients were considered to have undergone the urea breath test if the results were available in the medical records or if they reported having completed the test and provided the results (positive or negative) during the phone call. These results were used to calculate the eradication rate, defined as the proportion of negative tests among all available test results.

Patients for whom no follow-up informations were available in the medical records and who could not be contacted by phone or email (e.g., missing or invalid phone number, no response after three call attempts, or no email address) were classified as lost to follow-up.

### 2.4 Statistical analysis

Histological prevalence was expressed as percentages with 95% confidence intervals (95% CI). Quantitative variables were described using means and standard deviations for normally distributed data and medians with interquartile ranges for non-normally distributed data. Categorical variables were presented as numbers and percentages. Comparisons between groups were performed using the chi-square test or Fisher’s exact test, as appropriate.

### 2.5 Ethical and regulatory approval

This study was classified as Research Not Involving Human Participants (Recherche n’Impliquant pas la Personne Humaine, RnIPH) according to French regulations. As an internal research study using existing data, it complied with the requirements of the French National Data Protection Authority (Commission Nationale de l’Informatique et des Libertés, CNIL).

Participants were informed collectively through posters displayed in the Hepato-Gastroenterology Department, information provided in the hospital’s welcome booklet, and general information on clinical research available on the hospital website, indicating that research may be conducted using anonymized medical records and that patients have the right to refuse the use of their data.

## 3 Results

### 3.1 Prevalence and population characteristics

**Figure 1:**
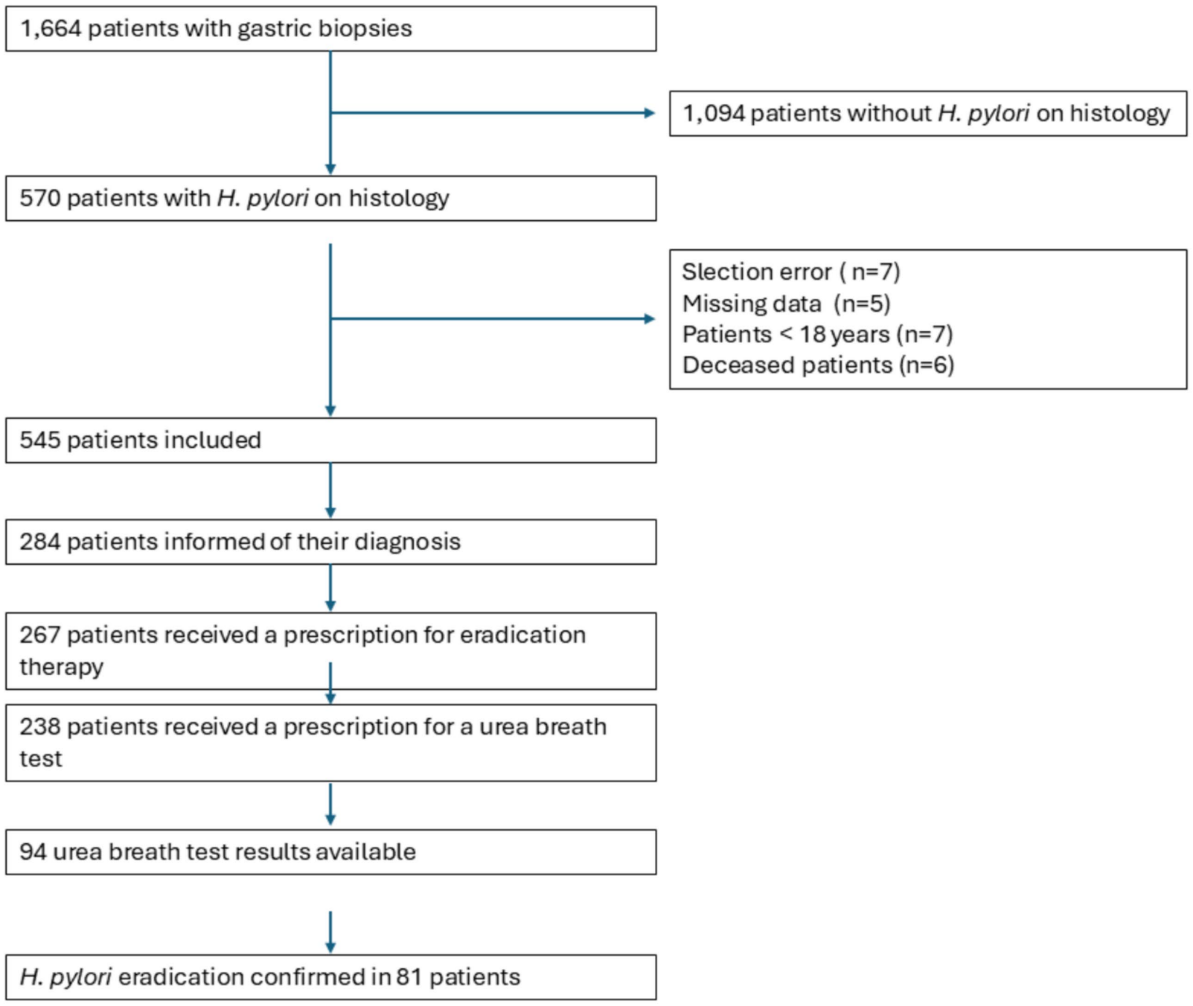
Flow chart.

Gastric biopsies for histological analysis were performed in 1,664 patients, of whom 570 tested positive for *H. pylori*. The histological prevalence of *H. pylori* infection was therefore 34.3% (95% CI 32.0–36.5). Among these 570 patients, 545 were included in the analysis (Figure 1).

The characteristics of the study population are summarized in Table 1. Women accounted for 57% of the population, and the mean age was 49.5 years. Approximately two-thirds of the patients were foreign-born.

**Table 1:** Patients’ characteristics

Variable	*N* (%)
Sex (*n* = 545)	
Male	236 (43)
Female	309 (57)
Age, mean (SD) (*n* = 545)	49.5 (±15.3)
Age groups, years (*n* = 545)	
18–30	61 (11)
31–50	229 (42)
>50	255 (47)
Place of birth (*n* = 543)	
France	204 (37.6)
Foreign	339 (62.4)
Health insurance (*n* = 545)	
State medical aid	100 (18.3)
Universal health coverage	90 (16.5)
Standard health insurance	337 (61.8)
No health insurance	18 (3.3)
Medical history	
Current smoking (*n* = 329)	82 (24.9)
Obesity (*n* = 370)	115 (31.1)
*H. pylori* infection (*n* = 459)	88 (19.2)

### 3.2 Indication for upper gastrointestinal endoscopy

**Figure 2:**
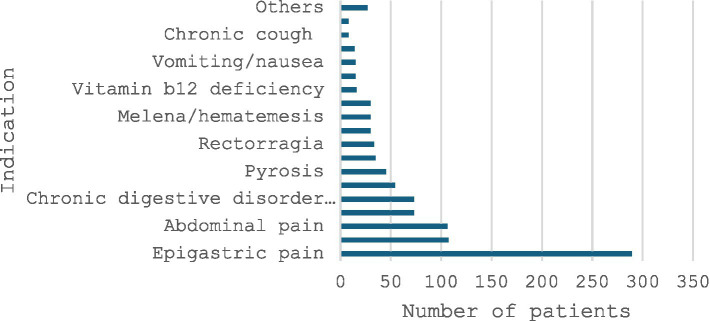
Indication for upper gastrointestinal endoscopy.

The main indications for UGE were epigastric pain (289 cases, 53.0%), gastroesophageal reflux disease (107 cases, 20%), and abdominal pain (106 cases, 19%) (Figure 2).

Women underwent UGE significantly more often than men for abdominal or epigastric pain, gastroesophageal reflux disease, pyrosis, and nausea or vomiting. Conversely, men underwent UGE significantly more often for upper gastrointestinal bleeding and rectorragia (Table 2).

**Table 2:** Indication for upper gastrointestinal endoscopy by sex.

Variable	Female (*n* = 309)	Male (*n* = 236)	*p* value*
Abdominal pain	70 (22.7)	36 (15.3)	**0.03**
Epigastric pain	178 (57.6)	111 (47.0)	**0.01**
Dyspeptic syndrome	19 (6.1)	11 (4.7)	0.45
Gastroesophageal reflux disease	73 (23.6)	34 (14.4)	**0.01**
Pyrosis	32 (10.4)	13 (5.5)	**0.04**
Chronic cough	2 (0.6)	6 (2.5)	0.08
Weight loss	39 (12.6)	34 (14.4)	0.54
Pre-bariatric surgery assessment	6 (1.9)	2 (0.8)	0.47
Family history of gastric cancer	10 (3.2)	5 (2.1)	0.43
Iron-deficiency anemia	33 (10.7)	21 (8.9)	0.49
Vitamin B12 deficiency	7 (2.3)	8 (3.4)	0.43
Melena or hematemesis	9 (2.9)	21 (8.9)	**0.002**
Rectorragia	13 (4.2)	20 (8.5)	**0.04**
Positive stool blood test	11 (3.6)	3 (1.3)	0.09
Altered general condition	19 (6.1)	16 (6.8)	0.76
Chronic digestive disorders (bloating, diarrhea, constipation)	80 (25.9)	47 (19.9)	0.10
Dysphagia	13 (4.2)	17 (7.2)	0.13
Nausea/vomiting	14 (4.5)	1 (0.4)	**0.004**

*Chi-square or Fisher’s exact test, as appropriate. Bold values indicate statistically significant differences (*p* < 0.05).

### 3.3 Endoscopic findings

**Figure 3:**
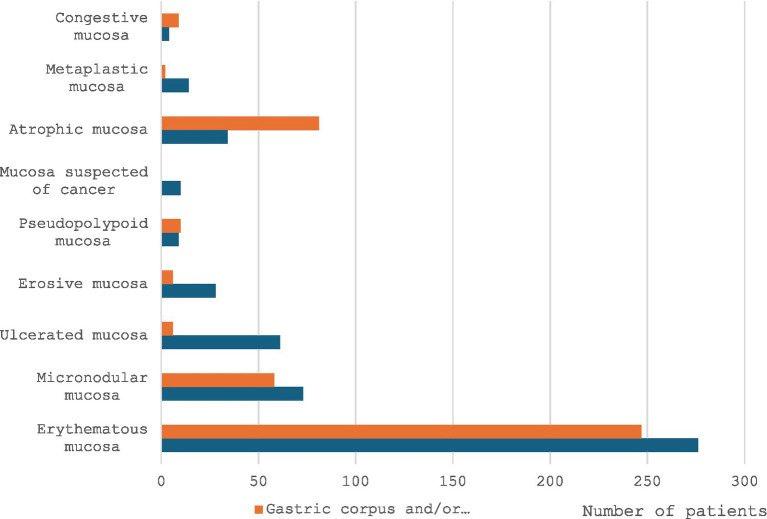
Endoscopic findings.

Upper gastrointestinal endoscopy was reported as normal in 21.6% of cases. The most frequent abnormality was erythematous gastritis, followed by an atrophic appearance and micronodular gastritis. Endoscopic findings are summarized in Figure 3.

### 3.4 Histological findings

*Helicobacter pylori* was detected simultaneously in the fundus and the antrum in the majority of cases (91.54%). Histological examination identified 9 cases of gastric cancer (adenocarcinoma) (1.65%).

Thirty-six patients had gastric atrophy (36/545, 6.60%), including 11 severe cases (11/545, 2.02%), estimated to correspond to OLGA stages 3–4 (Table 3).

**Table 3:** Histological analysis: number of patient with gastric atrophy.

*N* = 545	Corpus/fundus				
	Atrophy grade	None	Mild	Moderate	Severe
Antrum	None	509	3	4	0
	Mild	7	1	1	0
	Moderate	9	0	7*	0
	Severe	3*	0	0	1*

^*^ Patients classified as stage 3 or 4 according to the OLGA (operative link for gastritis assessment).

Regarding intestinal metaplasia, 105 patients (105/545, 19.30%) had intestinal metaplasia, including 11 extensive cases (11/545, 2.02%) estimated to correspond to OLGIM stage 3; no stage 4 cases were identified (Table 4).

**Table 4:** Histological analysis: number of patient with metaplasia

*N* = 545	Corpus/fundus			
	Intestinal metaplasia grade	None	Focal	Extensive
Antrum	None	440	9	1
	Focal	74	10	0
	Extensive	10*	1*	0

^*^ Patients classified as stage 3 according to the OLGIM (Operative Link on Gastric Intestinal Metaplasia).

Fourteen patients had dysplasia (14/545, 2.60%), including 11 cases of low-grade dysplasia and 3 cases of high-grade dysplasia (Table 5).

**Table 5:** Histological analysis: number of patient with gastric dysplasia

*N* = 545	Corpus/fundus			
	Dysplasia grade	None	Low-grade	High-grade
Antrum	None	531	0	0
	Low grade	8	3	0
	High grade	3	0	0

Overall, the proportion of precancerous lesions was 28.5%, and the proportion of severe precancerous lesions was 6.6%.

Finally, chronic gastritis was observed in 518 patients (518/545, 95.04%) (Table 6).

**Table 6:** Histological analysis: number of patient with chronic gastritis

*N* = 545	Corpus/fundus		
	Chronic gastritis	No	Yes
Antrum	No	27	9
	Yes	11	498

### 3.5 Follow-up analysis

At the time of data collection in May 2024 (more than four months after the last upper gastrointestinal endoscopy performed on December 31, 2023), 284 patients (52, 95% CI 47.8–56.3) were aware of their diagnosis, whereas 157 patients had not been informed. Information regarding awareness of the diagnosis was unavailable for 104 patients who were lost to follow-up.

A total of 267 patients (48, 95% CI 44.7–53.2) had been prescribed eradication therapy at the time of assessment (10-day bismuth quadruple therapy). Among them, 238 patients (89.1, 95% CI 81.8–90.4) were prescribed a urea breath test to assess eradication.

The therapy and the follow-up test were prescribed by a gastroenterologist in 77.2 and 87.4% of cases, respectively. Information on the results of the urea breath test was available for only 94 patients (94/238, 39.5%; 95% CI 34.5–47.8). Among these patients, the eradication rate was 86.2% (81/94).

**Figure 4:**
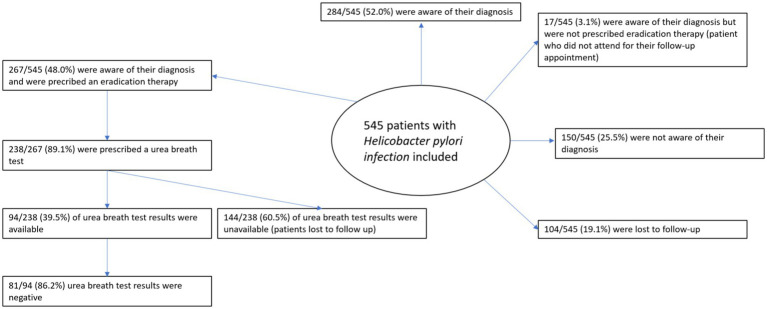
Follow-up outcomes.

Follow-up information was obtained from medical records in 40% of cases and from patient phone calls in another 40%. Overall, nearly 20% of patients (19.1, 95% CI 15.8–22.6) were lost to follow-up after UGE (Figure 4).

## 4 Discussion

This study found a histological prevalence of *H. pylori* infection of 34.3% in gastric biopsies. Severe precancerous lesions were identified in 6.6% of patients. Regarding follow-up, only 52% of patients were aware of their diagnosis at least three months after endoscopy, and 48% had received a prescription for eradication therapy.

The histological prevalence of *H. pylori* infection observed in our study was substantially higher than that reported in studies based on UGE in mainland France, where prevalence estimates are around 20% (15, 16). In a recent systematic review covering the period from 1980 to 2022, the prevalence of *H. pylori* infection in the Americas was estimated at 42.5% (95% CI 38.4–46.6), 39.6% (33.8–45.6) in Brazil, 44.8% (33.2–57.0) in Haiti, and 58.8% (53.2–64.1) in the Dominican Republic (17). However, in these studies, the diagnosis of *H. pylori* infection was not based solely on upper gastrointestinal endoscopy, as in our study, but also on serology or urea breath tests.

The main indications for UGE in our study were epigastric pain and gastroesophageal reflux disease, findings consistent with those reported by Hojati et al. (18). Similarly, erythematous or erosive gastritis was the most frequently described endoscopic abnormality. However, in our study only 20% of examinations were reported as normal, compared with 40% in their study.

The absence of an expert review of endoscopic images represents a limitation of this study. Indeed, variability in the description of endoscopic findings between operators may have introduced classification bias.

The proportion of precancerous lesions was 19.3% for intestinal metaplasia and 6.6% for gastric atrophy. In a study conducted in neighboring Brazil, intestinal metaplasia was reported in 17.7% of patients infected with *H. pylori*, and gastric atrophy in 17.6% (19). The proportion of severe precancerous lesions—defined as dysplasia or lesions estimated as stage 3–4 according to the OLGA and OLGIM classifications and requiring endoscopic surveillance—was 6.6%. We retrospectively estimated OLGA and OLGIM stages, which are widely recognized in international guidelines for gastric cancer risk stratification. Histological reports allowed reliable estimation of gastric atrophy, as it was classified as mild, moderate, or severe. However, intestinal metaplasia was classified as focal or extensive, with the latter grouping together moderate and severe forms. Consequently, the estimation of the OLGIM classification may be less precise and represents a limitation of this study. Our results for advanced atrophy and metaplasia (OLGA or OLGIM stages 3–4) were lower than those reported in a Korean cohort, where 25.8 and 14.5% of patients were classified as stage 3–4 according to the OLGA and OLGIM systems, respectively (20).

Our study has several limitations. First, the study population consisted of patients undergoing UGE, representing a selected subgroup of the general population that is more likely to present symptoms related to *H. pylori* infection. Nevertheless, this approach remains relevant for comparing prevalence with other studies using similar designs and provides the first estimates of *H. pylori* prevalence in this territory. Moreover, French Guiana experiences substantial immigration, with approximately one-third of the population being foreign-born (10), mainly from neighboring countries such as Brazil, Haiti, and other Caribbean territories where *H. pylori* prevalence is known to be high in the general population. Therefore, the prevalence observed in our study may reasonably reflect the underlying prevalence in the broader population.

Another limitation relates to the method used to detect infection. In this study, histological examination with immunohistochemistry was used. The main advantage of histology is the simultaneous characterization of gastric mucosal lesions. However, for the detection of *H. pylori*, histology is generally considered less sensitive and specific than molecular methods. Immunohistochemical analysis improves sensitivity, but molecular techniques such as *H. pylori* PCR provide higher sensitivity and specificity, even when patients have taken proton pump inhibitors during the two weeks preceding the examination (21). In our study, information on proton pump inhibitor or antibiotic use prior to upper gastrointestinal endoscopy was not available, which may have led to an underestimation of *H. pylori* prevalence. With the recent introduction of PCR testing in our center, the prevalence of *H. pylori* infection in gastric biopsies may be reassessed in future studies.

Finally, the proportion of patients lost to follow-up was high in this study. In addition to patients lost to follow-up immediately after UGE, some patients were also lost after being prescribed eradication therapy and the urea breath test. This represents a limitation in the assessment of follow-up outcomes. Although the eradication rate was high (86.2%) with bismuth quadruple therapy and consistent with previous reports (22), this result should be interpreted with caution because outcome data were available for only a limited number of patients.

Furthermore, although the recommended interval between discontinuation of antibiotics or proton pump inhibitors and performance of the urea breath test was clearly specified on the prescription document, strict adherence to these recommendations could not be verified.

To our knowledge, no published studies have specifically evaluated the follow-up of patients with a histological diagnosis of *H. pylori* infection. Our findings highlight important gaps in post-endoscopy management. Fewer than half of patients received a prescription for eradication therapy. This situation may partly be explained by barriers to healthcare access in this socioeconomically vulnerable population (13). Only a small proportion of patients were treated by their general practitioner, which may reflect delayed or insufficient communication between hospital specialists and primary care physicians. This lack of coordination in patient management may contribute to the high incidence of gastric cancer observed in French Guiana, although this hypothesis requires further investigation. Future studies should explore the factors associated with gastric cancer in this territory.

These findings underline the need to establish coordinated care pathways between specialists and primary care physicians in order to improve the management of *H. pylori* infection and reduce its long-term complications in French Guiana.

## 5 Conclusion

The histological prevalence of *Helicobacter pylori* infection in our population was 34.3%, which is higher than that reported in mainland France. The proportion of severe precancerous lesions requiring endoscopic surveillance was 6.6%. However, patient follow-up was inadequate, highlighting the need to improve eradication therapy and care coordination in order to reduce infection-related complications.

## Data Availability

Upon reasonable request and after approval from the Commission Nationale Informatique et Libertés (https://www.cnil.fr/fr/node/84666), data may be shared.
